# Feasibility study of personalized peptide vaccination for metastatic recurrent triple-negative breast cancer patients

**DOI:** 10.1186/bcr3685

**Published:** 2014-07-03

**Authors:** Ryuji Takahashi, Uhi Toh, Nobutaka Iwakuma, Miki Takenaka, Hiroko Otsuka, Mina Furukawa, Teruhiko Fujii, Naoko Seki, Akihiko Kawahara, Masayoshi Kage, Satoko Matsueda, Yoshito Akagi, Akira Yamada, Kyogo Itoh, Tetsuro Sasada

**Affiliations:** 1 Department of Surgery, Kurume University School of Medicine, 67 Asahi-machi, Kurume 830-0011, Japan; 2 Research Center for Innovative Cancer Therapy, Kurume University School of Medicine, 67 Asahi-machi, Kurume 830-0011, Japan; 3 Department of Pathology, Kurume University School of Medicine, 67 Asahi-machi, Kurume 830-0011, Japan; 4 Department of Immunology and Immunotherapy, Kurume University School of Medicine, 67 Asahi-machi, Kurume 830-0011, Japan

## Abstract

**Introduction:**

Since treatment modalities for metastatic recurrent triple-negative breast cancer (mrTNBC) are limited, a novel treatment approach including immunotherapy is required. We have developed a novel regimen of personalized peptide vaccination (PPV), in which vaccine antigens are individually selected from a pool of different peptide candidates based on the pre-existing host immunity. Herein we conducted a phase II study of PPV for metastatic recurrent breast cancer patients to investigate the feasibility of PPV for mrTNBC.

**Methods:**

Seventy-nine patients with metastatic recurrent breast cancer who had metastases and had failed standard chemotherapy and/or hormonal therapy were enrolled. They were subgrouped as the mrTNBC group (n = 18), the luminal/human epidermal growth factor receptor 2 (HER2)-negative group (n = 41) and the HER2-positive group (n = 18), while the remaining two patients had not been investigated. A maximum of four human leukocyte antigen (HLA)-matched peptides showing higher peptide-specific immunoglobulin G (IgG) responses in pre-vaccination plasma were selected from 31 pooled peptide candidates applicable for the four HLA-IA phenotypes (HLA-A2, -A24, or -A26 types, or HLA-A3 supertypes), and were subcutaneously administered weekly for 6 weeks and bi-weekly thereafter. Measurement of peptide-specific cytotoxic T lymphocyte (CTL) and IgG responses along with other laboratory analyses were conducted before and after vaccination.

**Results:**

No severe adverse events associated with PPV were observed in any of the enrolled patients. Boosting of CTL and/or IgG responses was observed in most of the patients after vaccination, irrespective of the breast cancer subtypes. There were three complete response cases (1 mrTNBC and 2 luminal/HER2-negative types) and six partial response cases (1 mrTNBC and 5 luminal/HER2-negative types). The median progression-free survival time and median overall survival time of mrTNBC patients were 7.5 and 11.1 months, while those of luminal/HER2-negative patients were 12.2 and 26.5 months, and those of HER2-positive patients were 4.5 and 14.9 months, respectively.

**Conclusions:**

PPV could be feasible for mrTNBC patients because of the safety, immune responses, and possible clinical benefits.

**Clinical Trial Registration Number:**

UMIN000001844 (Registration Date: April 5, 2009)

## Introduction

Recent advances in chemotherapies, hormonal therapies and anti-human epidermal growth factor receptor 2 (HER2) therapies have significantly improved the prognosis in metastatic recurrent breast cancer patients. For example, new chemotherapies using agents such as nanoparticle albumin-bound paclitaxel (nab-PTX) [1,2], eribulin mesylate [3,4] and bevacizumab [5-7], new hormonal therapies such as fluvestrant injection [8,9] or new anti-HER2 therapies such as those using pertuzumab [10,11] and trastuzumab emtansine (T-DM1) [12] have shown significant clinical benefits in metastatic recurrent breast cancer patients. Despite these novel therapeutic advances, the treatment modalities for chemotherapy-resistant triple-negative breast cancer (TNBC) remain limited, and thus a novel treatment approach including immunotherapy is required. Nevertheless, no randomized controlled trials of cancer vaccine have shown promise of clinical benefit for metastatic recurrent breast cancer patients, particularly in metastatic recurrent TNBC (mrTNBC).

We have developed a novel regimen of personalized peptide vaccination (PPV), in which vaccine antigens are selected from a pool of 31 different peptide candidates based on the pre-existing immunoglobulin G (IgG) responses specific to each peptide before vaccination [13-17]. Most of the peptides employed for PPV, except for those derived from prostate-related antigens, are known to be commonly expressed in various types of advanced cancers. Our previous clinical trials of PPV for patients with advanced cancers demonstrated the safety and feasibility of this new approach [13-17]. Here we conducted a phase II study of PPV for metastatic recurrent breast cancer to investigate the feasibility of PPV for mrTNBC.

## Methods

### Patients and methods

Women with a histological diagnosis of metastatic recurrent breast cancer were eligible for inclusion in the present study. All patients were required to have evaluable recurrent and/or metastatic tumors at the time of entry. Patients were divided into three different intrinsic subtypes as follows: luminal (estrogen-receptor-positive)/HER2-negative type, HER2-positive type (immunohistochemical score 3+ or HER2 gene/chromosome 17 ratio >2.2 in fluorescence *in situ* hybridization) and TNBC (hormone-receptor-negative and HER2-negative). Most patients had failed standard chemotherapy, but a few patients who had failed hormonal therapy alone were also eligible for this study. All patients were required to show positive IgG responses to at least 2 of the 31 different vaccine candidate peptides, as reported previously [13-17]. Other inclusion criteria were as follows: age between 20 and 80 years; an Eastern Cooperative Oncology Group (ECOG) performance status of 0 or 1; positive status for human leukocyte antigen (HLA)-A2, -A24 or -A26 types, or HLA-A3 supertypes (A3, A11, A31 or A33); life expectancy of at least 12 weeks; and adequate hematologic, hepatic and renal function. Exclusion criteria included pulmonary, cardiac or other systemic diseases; an acute infection; a history of severe allergic reactions; pregnancy or nursing; and other inappropriate conditions for enrollment as judged by clinicians. Patients with a lymphocyte count of <1,000/μL were excluded from the study, since we previously reported that pre-vaccination lymphocytopenia (<1,000 cells/μL) is an unfavorable factor for overall survival (OS) in cancer patients receiving PPV [17]. The protocol was approved by the Kurume University Ethical Committee and registered in the UMIN Clinical Trials Registry (Registration, number UMIN000001844; Registration date, 5 April 2009). All patients were given a full explanation of the protocol and provided their informed consent before enrollment.

### Clinical protocol

This was a phase II study to evaluate the safety and immunological responses in metastatic recurrent breast cancer patients under PPV. Thirty-one peptides, whose safety and immunological effects for other types of cancer were confirmed in previously conducted clinical studies [14-17], were employed for vaccination (12 peptides for HLA-A2, 16 peptides for HLA-A24, 9 peptides for HLA-A3 supertypes (-A3, -A11, -A31, and -A33) and 4 peptides for HLA-A26) (Additional file 1: Table S1). These peptides were prepared under the conditions of Good Manufacturing Practice (GMP) by the PolyPeptide Laboratories (San Diego, CA, USA) and American Peptide Company (Vista, CA, USA). Peptides for vaccination of individual patients were selected in consideration of the pre-existing host immunity before vaccination, as assessed by the titers of IgG specific to each of the 31 different vaccine candidates.

A maximum of four peptides (3 mg/each peptide), which were selected based on the results of HLA typing and peptide-specific IgG titers, were subcutaneously administered with incomplete Freund’s adjuvant (Montanide ISA51; Seppic, Paris, France) once a week for six consecutive weeks. After the first cycle of six vaccinations, up to four antigen peptides, which were re-selected according to the titers of peptide-specific IgG at the sixth vaccination, were administered every two weeks. After the second cycle of six vaccinations, up to four antigen peptides, which were also re-selected, were administered every four to eight weeks according to the immune responses after PPV. These protocols were continued until remarkable disease progression or disease in remission was shown, according to the will of the individual patient. During the PPV, patients were allowed to receive combination therapies such as chemotherapy, hormonal therapy, anti-HER2 therapy and radiotherapy. Adverse events were monitored according to the National Cancer Institute Common Terminology Criteria for Adverse Events version 3.0 (NCI-CTC Ver.-3.0). Complete blood counts and serum biochemistry tests were performed after every six vaccinations. The clinical responses were determined by the Response Evaluation Criteria in Solid Tumors (RECIST) in the vaccinated patients. The RECIST-based clinical responses were evaluated after nearly 12 vaccinations by radiological findings of computed tomography (CT) scan and/or magnetic resonance imaging (MRI), and the best overall responses during PPV treatment were shown. For the patients who did not complete the second cycle of 12 vaccinations, the newest radiological findings were evaluated, except in the case of patients who had died before the RECIST-based radiological evaluation.

### Measurement of humoral and cellular immune responses

Humoral immune responses specific to each of the 31 peptide candidates were determined by peptide-specific IgG levels using the Luminex system (Luminex, Austin, TX, USA), as previously reported [18]. If the titers of peptide-specific IgG to at least one of the vaccinated peptides in the post-vaccination plasma were more than two-fold higher than those in the pre-vaccination plasma, the changes were considered to be significant, as previously reported [14-17]. Cellular immune responses specific to the vaccinated peptides were evaluated by interferon (INF)-γ ELISPOT using peripheral blood mononuclear cells (PBMCs) as previously reported [14-17]. As a control, cellular immune responses specific to CEF peptides (MABTECH, Cincinnati, OH, USA), a mixture of virus-derived cytotoxic T lymphocyte (CTL) epitopes, were also examined.

### Statistical analyses

The Mann-Whitney U test and Fisher-Freeman-Halton exact test were used to examine statistical differences for continuous values and categorical values, respectively. *P*-values less than 0.05 were considered to be statistically significant. Progression-free survival (PFS) or OS was calculated from the date of the first vaccination until the date of disease progression or death, respectively, or the last date when the patient was known to be alive. The survival analysis was performed using the Kaplan-Meier method, and a comparison of the survival curves was performed with the log-rank test. Statistical tests were performed using JMP version 10 (SAS Institute Inc., Cary, NC, USA) and StatXact version 8 (Cytel Inc., Cambridge, MA, USA).

## Results

### Patient characteristics

Between January 2009 and April 2013, 79 metastatic recurrent breast cancer patients were enrolled in this study. The patient characteristics are shown in Table 1 for the overall patient group and each of the three subtypes. Among the 79 patients, 77 patients had been investigated to determine their intrinsic subtype before vaccination, while the remaining 2 patients had not. The HER2-positive group was associated with older median age (*P* = 0.019), restricted performance status (*P* = 0.046), higher positivity of HLA-A24 or -A2 (*P* = 0.039 or *P* = 0.015), higher frequency of visceral or brain metastasis (*P* = 0.009 or *P* = 0.002) and longer duration of previous chemotherapies (*P* <0.0001). Although the mrTNBC group had a shorter duration of previous chemotherapies (*P* <0.0001), most of the mrTNBC patients had received previous standard chemotherapy (anthracycline, *P* = 0.003; taxane, *P* = 0.0006).

**Table 1:** Comparison of patient characteristics for overall and breast cancer subtypes

**Character**	**Overall (number = 79)**	**mrTNBC (number = 18)**	**Luminal/HER2-negative (number = 41)**	**HER2-positive (number = 18)**	***P*-value**^**a**^
Median age (range)	57 (30 to 77)	55 (30 to 65)	55 (39 to 76)	62 (51 to 70)	0.019
Performance status					0.046
0/1	58/21	14/4	32/9	11/7	
Median time to the first PPV from recurrence, months (range)	35 (1.2 to 165)	8 (1.2 to 99)	40 (2 to 123)	55 (19 to 165)	0.101
Histopathology					0.079
Ductal carcinoma	71	16	37	17	
Lobular carcinoma	4	1	2	1	
Others	4	1	2		
Positive status of HLA-A24	55	10	29	14	0.039
Positive status of HLA-A2	22	6	8	8	0.015
Visceral metastasis					0.009
Yes/No	60/19	8/6	29/11	16/2	
Brain metastasis					0.002
Yes/No	11/68	3/11	2/38	6/12	
Median duration of previous chemotherapies, months (range)	12 (2 to 148)	9 (4 to 43)	12 (2 to 9)	37 (10 to 148)	<0.0001
Usage of previous standard chemotherapy					
Anthracycline	50	16	22	12	0.003
Taxane	58	16	24	17	0.0006
Trastuzumab	18		3	15	<0.0001
Regimen number of previous chemotherapies					0.008
<4/≥4	36/36	8/9	22/14	6/12	
Combined therapies					
Oral chemotherapy	19	5	8	6	0.060
Infusion chemotherapy	32	10	15	7	0.038
Anti-HER2 therapy	11			11	<0.0001
Hormonal therapy	23	2	17	4	0.006
Median times of peptide vaccination	14 (2 to 39)	12 (2 to 30)	15 (4 to 39)	12 (6 to 22)	0.021

^a^The Mann-Whitney U test and Fisher-Freeman-Halton exact test were performed to examine *P*-values for continuous values and categorical values. HER2, human epidermal growth factor 2; HLA, human leukocyte antigens; mrTNBC, metastatic recurrent triple-negative breast cancer; PPV, personalized peptide vaccination.

### Combined therapies and adverse events

The median number of peptide vaccinations was 14, with a range from 2 to 39 vaccinations (Table 1). Table 2 shows all adverse events during the PPV. As the vaccination-related adverse events, all patients showed grade 1 or 2 dermatological reactions to PPV at the injection sites, but no patients showed severe adverse events (grade 3 or more). Forty-one patients (52.0%) showed grade 3 or 4 adverse events strongly associated with combined chemotherapies and disease progression (Table 2).

**Table 2:** Adverse events during the PPV

**Adverse event**	**Grade 1**	**Grade 2**	**Grade 3**	**Grade 4**
Injection site reaction	42	37		
Constitutional symptom				
Fever	8	1		
Malaise	7	3		
Edema limbs	2	3		
Pain	5			
Tumor pain	4	9		
Gastrointestinal				
Nausea	4			
Mucositis oral		1		
Abdominal pain			1	
Constipation	1	1		
Diarrhea	2			
Respiratory				
Dyspnea	5	1	1	
Cough	5			
Hoarseness	2			
Pneumonitis		1		
Pleural effusion	1			
Hypoxia		1	2	
Neurological				
Headache	1	1		
Dysgeusia	1			
Dizziness	2	1		
Peripheral sensory neuropathy	8	2		
Peripheral motor neuropathy	1			
Endocrine disorder				
Hypothyroidism		1		
Skin and subcutaneous				
Pruritis	33	3		
Urticaria	4	1		
Reproductive system				
Vaginal hemorrhage	2			
Vascular disorders				
Hot flashes	1			
Lymphedema		1		
Hypertension		1		
Blood/Bone marrow				
Anemia	18	9	2	2
Hemoglobin increased	1			
Leukocytopenia	22	11	4	
Neutropenia	2	6	5	1
Lymphocytopenia	29	7	12	
Thrombocytopenia	6		1	
Metabolism and nutrition				
Anorexia	2			
Hyponatremia	1			
Hyperkalemia	3			
Hypocalcemia	2			
Hyperglycemia	2			
Laboratory				
AST increased	14	9	2	
ALT increased	19	4	2	
γ-GTP increased	7	3	2	
ALP increased	6	1	1	
Hyperbilirubinemia	3	1	1	
Creatinine increased	10	3	1	
Cholesterol high	4			
Hypoalbuminemia	46	7		
INR increased	1	1	1	
APTT increased	1			

ALP, alkaline phosphatase; ALT, alanine aminotransferase; AST, aspartate aminotransferase; APTT, activated partial thromboplastin time; γ-GTP, gamma-glutamyl transpeptidase; INR, international normalized ratio; PPV, personalized peptide vaccination.

During the PPV, 51 patients (64.6%; 32 cases with infusion chemotherapy and 19 cases with oral chemotherapy) received combined chemotherapies, while 23 patients (29.1%) and 11 patients (13.9%) received hormonal therapies and anti-HER2 therapies, respectively (Table 1). The mrTNBC patients received combined infusion chemotherapy more frequently than other breast cancer subtypes (*P* = 0.038). The most commonly used chemotherapy drug was capecitabine (fifteen cases; 19.0%), followed by gemcitabine (eight cases; 10.1%), eribulin mesylate (six cases; 7.6%), FEC (5-fluorouracil, epirubicin and cyclophosphamide), nab-PTX or vinorelbine (four cases each; 5.1%), paclitaxel (three cases; 3.8%), bevacizumab, irinotecan or S-1 (two cases each; 2.5%), and docetaxel, oral cyclophosphamide or tegafur (one case each; 1.3%). Eleven patients (13.9%) received combined anti-HER2 therapies including trastuzumab (six cases; 7.6%) and lapatinib (five cases; 6.3%); combined anti-HER2 therapy was the most used treatment for the HER2-positive group (*P* <0.0001). In addition, 23 patients (29.1%) received combined hormonal therapies using agents, such as aromatase inhibitor (16 cases; 20.3%), high-dose toremifene (5 cases; 6.3%) and fluvestrant (2 cases; 2.5%); combined hormonal therapy was the most used treatment for the luminal/HER2-negative group (*P* = 0.046).

### Immune responses to the vaccinated peptides

Both humoral and cellular immune responses specific to the vaccinated peptides were analyzed in blood samples before and after vaccination. Plasma samples were collected from 79, 75, or 53 patients before vaccination, at the 6th vaccination, or at the 12th vaccination, respectively. For the monitoring of humoral immune responses, peptide-specific IgGs reactive to each of the 31 different peptides, including both vaccinated and non-vaccinated peptides, were measured by bead-based multiplex assay. The numbers of peptides employed for the first cycle of vaccinations were 2, 3, or 4 in 8, 6, or 63 patients, respectively (Additional file 2: Table S2, Additional file 3: Table S3, and Additional file 4: Table S4). Augmentation of IgG responses specific to at least one of the vaccinated peptides after 6 or 12 vaccinations was observed in 53/75 (70.7%) patients or 50/53 (94.3%) patients, respectively. Peptide-specific IgG responses after 6 or 12 vaccinations were augmented in 7/15 (46.7%) patients or 9/10 (90%) patients with mrTNBC (Additional file 2: Table S2). Such augmentation was seen in 28/40 (70.0%) patients or 29/31 (93.5%) patients in the luminal/HER2-negative group (Additional file 3: Table S3) and in 16/18 (88.9%) patients or 11/11 (100%) patients of the HER2-positive group (Additional file 4: Table S4), respectively.

Cellular immune responses to vaccinated peptides were assessed by IFN-γ ELISPOT assay. Antigen-specific CTL responses were detectable in 17/66 (25.8%) patients before vaccination (Additional file 2: Table S2, Additional file 3: Table S3 and Additional file 4: Table S4). In contrast, augmentation of the CTL responses specific to at least one of the vaccinated peptides after six vaccinations was observed in 34/63 patients (54.0%). Peptide-specific CTL responses after six vaccinations were augmented in 7/14 (50.0%) patients with mrTNBC (Additional file 2: Table S2), while such augmentation was seen in 18/31 (58.1%) patients and 7/16 (43.8%) patients in the luminal/HER2-negative group (Additional file 3: Table S3) and HER2-positive group (Additional file 4: Table S4), respectively. We also tested CTL responses to CEF peptides, a mixture of virus-derived CTL epitopes, as a control. CTL responses to CEF peptides were observed in 27/62 (43.5%) patients before vaccination and 15/58 (25.9%) patients after six vaccinations (Additional file 2: Table S2, Additional file 3: Table S3 and Additional file 4: Table S4).

Collectively, 30/63 (47.6%) patients showed both increased CTL and IgG responses to the vaccinated peptides, 23/63 (36.5%) patients showed either increased CTL or IgG responses, and the remaining 10 (15.9%) patients showed neither CTL nor IgG boosting. In patients treated with PPV alone (n = 27), IgG responses were more frequently increased than those in patients treated with combined chemotherapies (n = 47) (*P* = 0.002), although there was no significant difference in the increase in CTL responses (*P* = 1.000).

### Clinical responses to PPV

**Figure 1:**
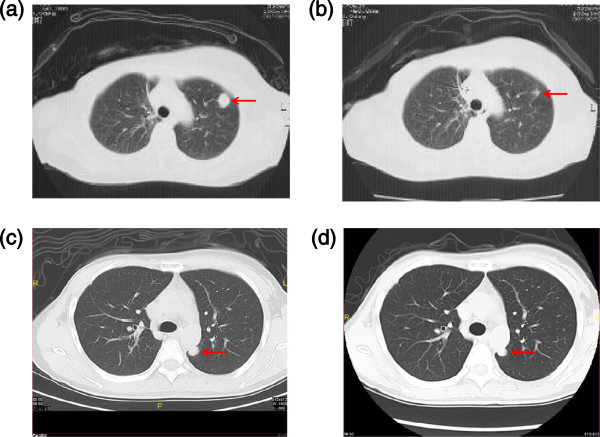
**Clinical responses to PPV. a, b)** Computed tomography findings of a PR case with mrTNBC (case 2 in Table S2) before and after the 12th vaccination. A 63-year-old woman with a recurrent lung mass underwent 12 vaccinations in combination with gemcitabine (1,000 mg/m^2^/week for three weeks followed by one week intermission). At four months after the first vaccination, the lung mass was remarkably reduced in size (arrow). **c, d)** Computed tomography findings of a SD case with mrTNBC (case 18 in Table S2) before and after the eighth vaccination. A 34-year-old woman with a recurrent lung mass underwent eight vaccinations in combination with two cycles of eribulin mesylate (1.4 mg/m^2^/week for two weeks followed by one week intermission). At three months after the first vaccination, the lung mass was slightly decreased in size (arrow). mrTNBC, metastatic recurrent triple negative breast cancer; PPV, personalized peptide vaccination; PR, partial response; SD, stable disease.

**Figure 2:**
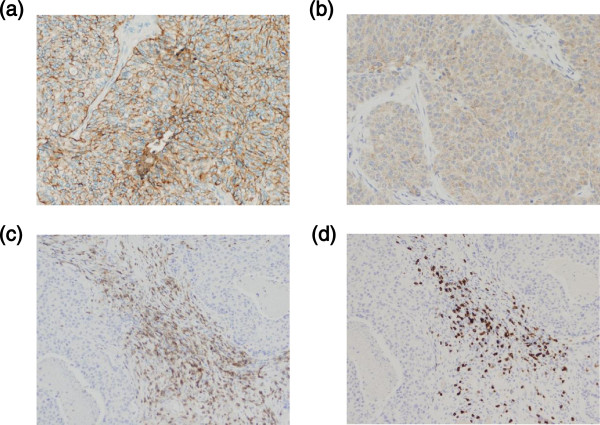
**Expressions of TAAs and pathological responses to PPV.** After completion of eight vaccinations in combination with two cycles of eribulin mesylate (1.4 mg/m^2^/week for two weeks followed by one week intermission), the lung metastasis of a SD case with mrTNBC (case 18 in Table S2) was resected at three months after the first vaccination. The TAA expression and T cell infiltration in the resected lung tissue were examined by immunohistochemistry. **a, b)** Among the four TAAs, that is, SART2, PSA, EGF-R and LCK, two TAAs were expressed in the lung tumor. **a)** EGF-R (X200); **b)** SART2 (X200). **c, d)** Peritumoral infiltration of T lymphocytes was confirmed in the lung tumor. **c)** CD4^+^ T lymphocytes (X200); **d)** CD8+ T lymphocytes (X200). EGF-R, epidermal growth factor receptor; LCK, lymphocyte specific protein tyrosine kinase; mrTNBC, metastatic recurrent triple negative breast cancer; PPV, personalized peptide vaccination; PSA, prostate specific antigen; SART2, squamous cell carcinoma antigen recognized by T-cells 2; SD, stable disease; TAA, tumor associated antigens.

The RECIST-based clinical responses were evaluated in 64 patients by radiological findings. There were 3 complete response (CR), 6 partial response (PR), 27 stable disease (SD) and 28 progressive disease (PD). The overall response rate of PPV was 14%, including three CR and six PR cases. Among the responsive patients, combined chemotherapy was used in eight cases and hormonal therapy in one case. The intrinsic subtypes showed one mrTNBC and two luminal/HER2-negative types in the CR cases and one mrTNBC and five luminal/HER2-negative types in the PR cases. Computed tomography findings of each of the mrTNBC cases showing PR or SD are shown in Figure 1. The PR case (case 2 in Additional file 2: Table S2) was a 63-year-old woman with a recurrent lung mass treated with a combination of gemcitabine and PPV. At four months after the first vaccination, the lung mass was remarkably reduced in size (Figure 1a and Figure 1b). She survived 32 months after the first vaccination and died due to disease progression. The SD case (case 18 in Additional file 2: Table S2) was a 34-year-old woman with a recurrent lung mass treated with a combination of eribulin mesylate and PPV. At three months after the first vaccination, the lung mass was slightly decreased in size (Figure 1c and Figure 1d). She was subsequently treated by radical resection of the lung mass and pathological evaluation. The lung mass was metastatic TNBC with a high Ki-67 labeling index (42.0%). It expressed epidermal growth factor receptor (EGF-R) and squamous cell carcinoma antigen recognized by T-cells 2 (SART2) antigens which were vaccinated antigens (Figure 2a and Figure 2b), and peritumoral infiltration of T lymphocytes was confirmed (Figure 2c and Figure 2d). She is still alive at 13 months following the first vaccination.

### Survival analyses by intrinsic subtypes

**Figure 3:**
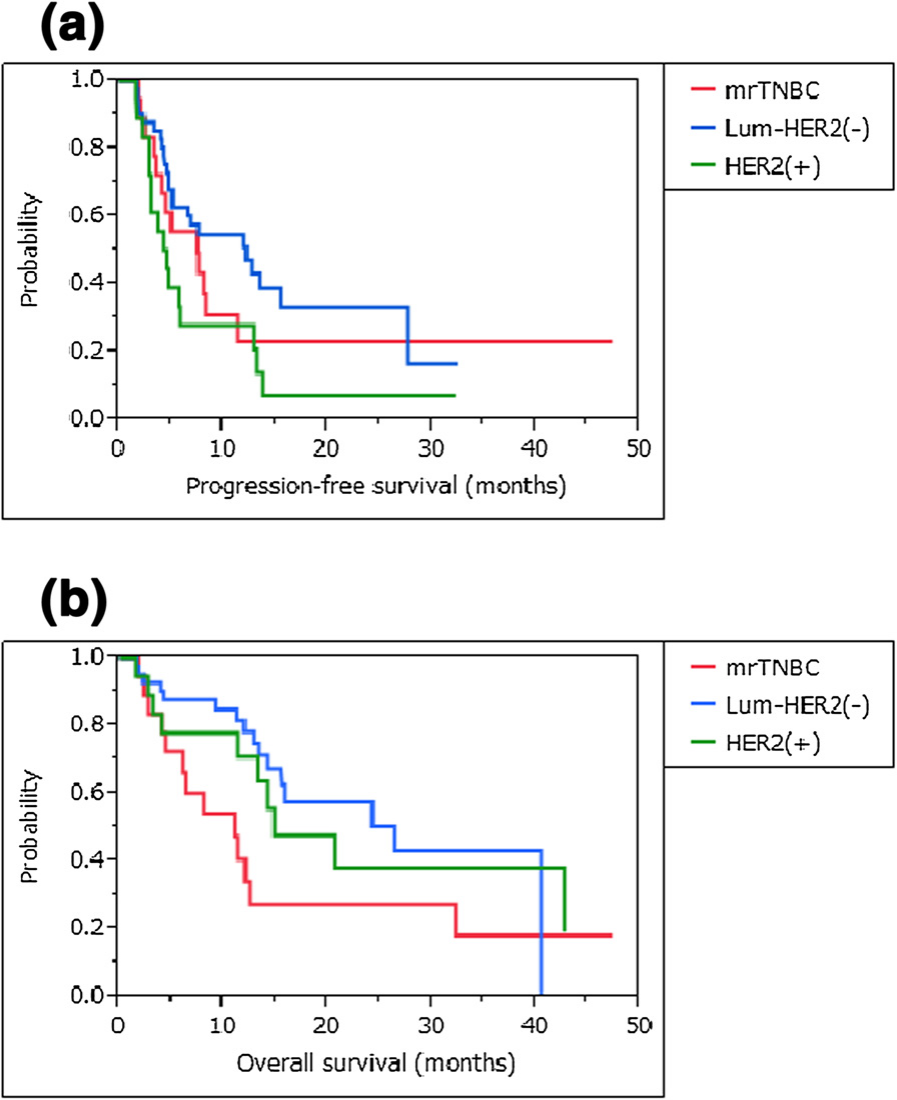
**Survival curves among the three intrinsic subtypes. a)** The median progression-free survival time of mrTNBC patients was 7.5 months, while that of luminal/HER2-negative patients was 12.2 months, and that of HER2-positive patients was 4.5 months. **b)** The median overall survival time of mrTNBC patients was 11.1 months, while that of luminal/HER2-negative patients was 26.5 months, and that of HER2-positive patients was 14.9 months. HER2, human epidermal growth factor receptor 2; mrTNBC, metastatic recurrent triple negative breast cancer.

**Figure 4:**
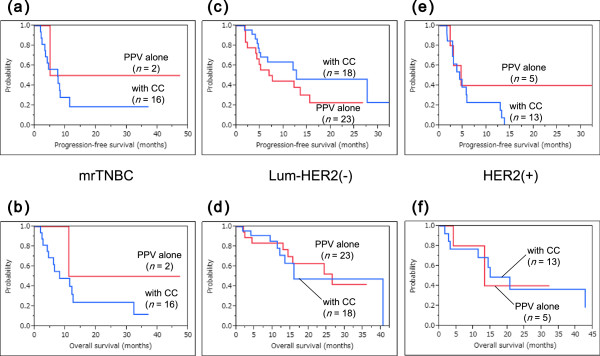
**Survival curves for patients treated with PPV with or without combination chemotherapies. a-f)** There was no significant survival advantage of combined chemotherapies (CC) in each intrinsic subtype, compared with the treatment by PPV alone. **a) b)** mrTNBC: PFS and OS, *P* = 0.467 and *P* = 0.347, respectively. **c,d)** luminal/HER2-negative type: PFS and OS, *P* = 0.220 and *P* = 0.850, respectively. **e,f)** HER2-positive type: PFS and OS, *P* = 0.296 and *P* = 0.957, respectively. HER2, human epidermal growth factor receptor 2; mrTNBC, metastatic recurrent triple negative breast cancer; OS, overall survival; PFS, progression-free survival; PPV, personalized peptide vaccination.

Figure 3 shows survival curves for the three intrinsic subtypes. The median progression-free survival time (MPFST) and median overall survival time (MST) of mrTNBC patients were 7.5 and 11.1 months, while those of luminal/HER2-negative patients were 12.2 and 26.5 months, and those of HER2-positive patients were 4.5 and 14.9 months, respectively. For each intrinsic subtype, the survival curves were compared for patients treated with PPV plus concurrent chemotherapies and those treated with PPV alone (Figure 4). There was no significant survival advantage of concurrent chemotherapies in each intrinsic subtype, compared with treatment with PPV alone (mrTNBC: PFS and OS, *P* = 0.467 and *P* = 0.347, respectively; luminal/HER2-negative type: PFS and OS, *P* = 0.220 and *P* = 0.850, respectively; and HER2-positive type: PFS and OS, *P* = 0.296 and *P* = 0.957, respectively).

**Figure 5:**
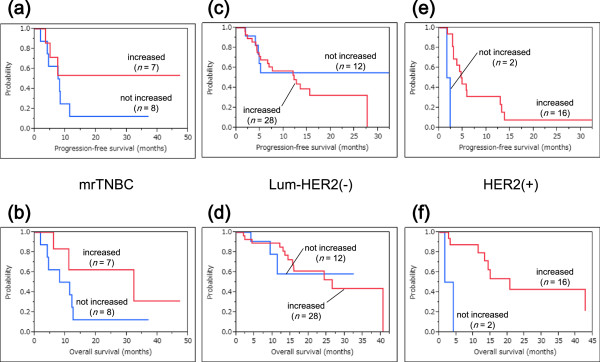
**Survival curves for patients with or without increased IgG responses after PPV. a-f)** IgG boosting was a significant prognostic factor for OS and PFS in HER2-positive patients, whereas there was no significant difference between increased IgG responses and these prognoses in mrTNBC and luminal/HER2-negative patients. **a, b)** mrTNBC: PFS and OS, *P* = 0.274 and *P* = 0.152, respectively. **c, d)** luminal/HER2-negative type: PFS and OS, *P* = 0.732 and *P* = 0.571, respectively. **e, f)** HER2-positive type: PFS and OS, *P* = 0.0001 and *P* = 0.001, respectively. HER2, human epidermal growth factor receptor 2; IgG, immunoglobulin G; mrTNBC, metastatic recurrent triple negative breast cancer; OS, overall survival; PFS, progression-free survival; PPV, personalized peptide vaccination.

**Figure 6:**
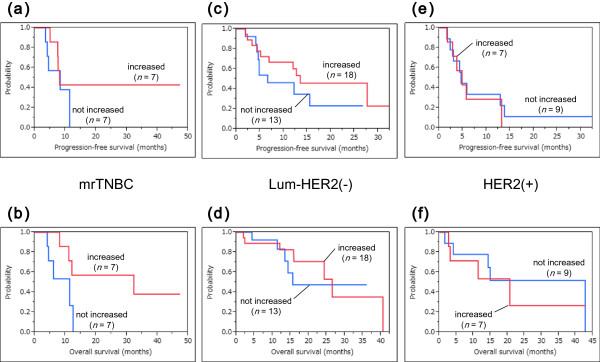
**Survival curves for patients with or without increased CTL responses after PPV. a-f)** CTL boosting was suggested to be a potential prognostic factor for OS but not for PFS in mrTNBC patients, whereas there was no significant difference between CTL boosting and these prognoses in luminal/HER2-negative and HER2-positive patients. **a, b)** mrTNBC: PFS and OS, *P* = 0.345 and *P* = 0.053, respectively. **c, d)** luminal/HER2-negative type: PFS and OS, *P* = 0.272 and *P* = 0.740, respectively. **e, f)** HER2-positive type: PFS and OS, *P* = 0.714 and *P* = 0.758, respectively. CTL, cytotoxic T lymphocytes; HER2, human epidermal growth factor receptor 2; mrTNBC, metastatic recurrent triple negative breast cancer; OS, overall survival; PFS, progression-free survival; PPV, personalized peptide vaccination.

Survival analyses along with analyses of the immune responses to PPV were also conducted in the three subtypes. Figure 5 shows the survival curves for patients with or without increased IgG responses after PPV in each intrinsic subtype. IgG boosting was a significant prognostic factor for OS and PFS in HER2-positive patients, whereas there was no significant difference between increased IgG responses and these prognoses in mrTNBC and luminal/HER2-negative patients (mrTNBC: PFS and OS, *P* = 0.274 and *P* = 0.152, respectively; luminal/HER2-negative type: PFS and OS, *P* = 0.732 and *P* = 0.571, respectively; HER2-positive type: PFS and OS, *P* = 0.0001 and *P* = 0.001, respectively). Figure 6 shows the survival curves for patients with or without increased CTL responses after PPV in each intrinsic subtype. CTL boosting was suggested to be a potential prognostic factor for OS but not for PFS in mrTNBC patients, whereas there was no significant difference between CTL boosting and these prognoses in luminal/HER2-negative and HER2-positive patients (mrTNBC: PFS and OS, *P* = 0.345 and *P* = 0.053, respectively; luminal/HER2-negative type: PFS and OS, *P* = 0.272 and *P* = 0.740, respectively; HER2-positive type: PFS and OS, *P* = 0.714 and *P* = 0.758, respectively).

## Discussion

Since treatment outcomes in mrTNBC patients remain poor [19-21], a novel treatment modality including immunotherapy is required. Several tumor associated antigens (TAAs), such as cancer testis antigens, EGF-R, aldehyde dehydrogenase 1 (ALDH1) and enhancer of zeste homolog 2 (EZH2), are frequently expressed in TNBC, particularly in basal-like subtypes [22-24]. Despite these potential molecular targets for immunotherapy in TNBC, no randomized controlled trials of cancer vaccine have shown promise of clinical benefit to date. We have developed a novel regimen of PPV, in which vaccine antigens are selected and administered from a pool of 31 different peptide candidates based on the pre-existing IgG responses specific to peptides before vaccination [13-17]. In previous studies, PPV was feasible for the vast majority of cancer patients with different HLA-types [13-17]. Based on these results in cancer patients, we conducted a phase II study of PPV for metastatic recurrent breast cancer patients to investigate the feasibility of PPV for mrTNBC. There were no severe adverse events associated with PPV, and most of the mrTNBC patients showed augmented immune responses to PPV.

The current study suggested the feasibility of PPV for mrTNBC patients who had failed standard chemotherapy, since the MPFST and MST of mrTNBC patients were 7.5 and 11.1 months from the first vaccination, respectively. In previously reported studies, the MPFST of mrTNBC patients treated by various chemotherapy and/or targeted therapy regimens was between 2.5 and 6.5 months [7,25-28]. Therefore, the MPFST of 7.5 months in mrTNBC patients treated by PPV in the current study seemed to be promising. Regarding OS in TNBC patients, Dent *et al*. demonstrated that the MST from recurrence to death was nine months, although the details of chemotherapy regimens were not described [19]. More recently conducted studies showed that the MST of mrTNBC patients treated by various chemotherapy and/or targeted therapy regimens was between 7.7 and 17.9 months [26-28]. Although almost all patients in these previous studies were enrolled as a first-line and/or second-line treatment [26-28], most patients in the current study were enrolled as a third or more line treatment. In addition, considering that the median duration of previous chemotherapies in the mrTNBC patients in the current study was 9 months, the MST of 11.1 months in mrTNBC patients treated by PPV seems to be encouraging. As a next step, to clarify the clinical benefit of PPV in mrTNBC, we need to conduct a randomized controlled study, in which patients are treated with standard of care (SOC) alone or with PPV plus SOC.

Although the results of immune responses were not significantly different by intrinsic subtypes, a high population of HER2-positive patients showed IgG responses at the sixth vaccination. Since all of the HER2-positive patients had been treated with trastuzumab, antigen-dependent cellular cytotoxicity might have affected their humoral immunities [29]. Notably, IgG boosting was a significant prognostic factor for OS and PFS in HER2-positive patients, although the number of patients was too small to confirm this. Combined chemotherapies also might affect the status of IgG responses, but no survival advantages of combined chemotherapies were shown in our metastatic recurrent breast cancer patients.

The clinical response rate in the present series was 14.0%, including three CR and six PR cases. Among these responsive patients, combined chemotherapy was used in eight cases and hormonal therapy in one case. The intrinsic subtype of these patients was luminal/HER2-negative type in seven cases and mrTNBC in two cases. Notably, the number of previous chemotherapy regimens was one regimen in two patients and two regimens in seven patients. In our study, more than four regimens of previous chemotherapy were significantly correlated with poor prognosis (data not shown). From these results, we would recommend PPV within three regimens of previous chemotherapy for metastatic recurrent breast cancer patients. A greater number of previous chemotherapy regimens could not sufficiently enhance clinical responses to PPV, particularly in HER2-positive patients. Because of the significant clinical benefit and conventional usage of trastuzumab, the duration of previous chemotherapy was significantly prolonged in HER2-positive patients. The status of tumor molecular biology could change and be complicated by this long-term chemotherapy, eventually leading to a poor prognosis. For HER2-positive patients, the induction of PPV should be earlier than that in our HER2-positive patients. Since combined chemotherapies increase the number of severe adverse events in metastatic breast cancer patients, treatment with PPV alone should be performed to maintain their quality of life.

We had an opportunity to confirm the peritumoral infiltration of lymphocytes in the lung metastasis of mrTNBC. Two of the four vaccinated peptide antigens (EGF-R and squamous cell carcinoma antigen recognized by T-cells 2 (SART2)) were expressed in the lung tumor, and CTL responses to the SART2-93 antigen were significantly increased in this case (case 18 in Additional file 2: Table S2). Although IgG responses to the two peptide antigens were not significantly increased, PPV could enhance the anti-tumor immunity and efficacy of combined chemotherapy in this case. We have investigated the expressions of 15 TAAs in primary and recurrent breast cancer tissues by immunohistochemistry (RT, unpublished data). We found that 10 of 15 TAAs were expressed in both primary and recurrent breast cancer tissues, except for lymphocyte specific protein tyrosine kinase (LCK), prostate specific antigen (PSA), prostate specific membrane antigen (PSMA), prostatic acid phosphatase (PAP) and multidrug resistance-associated protein 3 (MRP3). However, four of these five TAAs, including LCK, PSA, PAP and MRP3, were reported to be expressed in breast cancer tissues, although the frequency of expression was lower than that of other TAAs [30-34]. Therefore, 14 of the 15 TAAs could be potential molecular targets for immunotherapy in breast cancer patients.

## Conclusions

In conclusion, PPV could be feasible for mrTNBC patients because of the safety, immune responses and possible clinical benefits. For mrTNBC patients, we are planning a randomized controlled study, in which patients are treated with SOC alone or with PPV plus SOC to further clarify the clinical benefit of PPV.

## Abbreviations

ALDH1: aldehyde dehydrogenase 1; CR: complete response; CT: computed tomography; CTL: cytotoxic T lymphocyte; ECOG: Eastern Cooperative Oncology Group; EGF-R: epidermal growth factor receptor; EZH2: enhancer of zeste homolog 2; FEC: 5- fluorouracil/epirubicin/cyclosphamide; HER2: human epidermal growth factor 2; INF: interferon; LCK: lymphocyte specific protein tyrosine kinase; MPFST: median progression-free survival time; MRI: magnetic resonance imaging; MRP3: multidrug resistance-associated protein 3; mrTNBC: metastatic recurrent triple-negative breast cancer; MST: median overall survival time; nab-PTX: nanoparticle albumin-bound paclitaxel; NCI-CTC Ver.-3.0: National Cancer Institute common terminology criteria for adverse events version 3.0; OS: overall survival; PAP: prostatic acid phosphatase; PBMCs: peripheral blood mononuclear cells; PD: progressive disease; PFS: progression-free survival; PPV: personalized peptide vaccination; PR: partial response; PSA: prostate specific antigen; PSMA: prostate specific membrane antigen; RECIST: Response Evaluation Criteria in Solid Tumors; SART2: squamous cell carcinoma antigen recognized by T-cells 2; SD: stable disease; SOC: standard of care; TAAs: tumor associated antigens; T-DM1: trastuzumab emtansine; TNBC: triple-negative breast cancer.

## Competing interests

Akira Yamada, is a Board member of the Green Peptide Company, Ltd. Kyogo Itoh and Akira Yamada have stock of the Green Peptide Company, Ltd. Kyogo Itoh received research fund from Taiho Pharmaceutical Company. The other authors declare that they have no competing interests.

## Authors’ contributions

RT, UT and KI are responsible for the conception and design of the study, the acquisition, analysis and interpretation of data, and drafting the work. TS is responsible for the interpretation of data and drafting the work. NI, MT, HO, MF, TF, NS, YA and AY are responsible for the interpretation of data and revising the work critically. AK, MK and SM are responsible for the acquisition and analysis of data and revising the work critically. All authors read and approved the final manuscript.

## Supplementary Material

Additional file 1: Table S1 Information on the peptide candidates used for PPV. Click here for file

Additional file 2: Table S2 Immune responses to vaccinated peptides in mrTNBC patients. Click here for file

Additional file 3: Table S3 Immune responses to vaccinated peptides in luminal/HER2-negative patients. Click here for file

Additional file 4: Table S4 Immune responses to vaccinated peptides in HER2-positive patients. Click here for file
