# Supplementary material for: Feasibility study of personalized peptide vaccination for metastatic recurrent triple-negative breast cancer patients
Source: Breast Cancer Res. 2014 Jul 3;16(4):R70. doi: 10.1186/bcr3685 (PMC4227005; doi:10.1186/bcr3685)
Supplement: Additional file 1: Table S1 — Information on the peptide candidates used for PPV. [file bcr3685-S1.docx]

**Table S1.　Information on the peptide candidates used for PPV**

| Peptide name | Original protein | Position | Sequence | HLA-IA restriction | References |
| --- | --- | --- | --- | --- | --- |
| CypB-129  Lck-246  Lck-422  ppMAPkkk-432  WHSC2-103  HNRPL-501  UBE2V-43  UBE2V-85  WHSC2-141  HNRPL-140  SART3-302  SART3-309  SART2-93  SART3-109  Lck-208  PAP-213  PSA-248  EGF-R-800  MRP3-503  MRP3-1293  SART2-161  Lck-486  Lck-488 | Cyclophilin B  p56 ^lck^  p56 ^lck^  ppMAPkkk  WHSC2  HNRPL  UBE2V  UBE2V  WHSC2  HNRPL  SART3  SART3  SART2  SART3  p56 ^lck^  PAP  PSA  EGF-R  MRP3  MRP3  SART2  p56 ^lck^  p56 ^lck^ | 129-138  246-254  422-430  432-440  103-111  501-510  43-51  85-93  141-149  140-148  302-310  309-317  93-101  109-118  208-216  213-221  248-257  800-809  503-511  1293-1302  161-169  486-494  488-497 | KLKHYGPGWV  KLVERLGAA  DVWSFGILL  DLLSHAFFA  ASLDSDPWV  NVLHFFNAPL  RLQEWCSVI  LIADFLSGL  ILGELREKV  ALVEFEDVL  LLQAEAPRL  RLAEYQAYI  DYSARWNEI  VYDYNCHVDL  HYTNASDGL  LYCESVHNF  HYRKWIKDTI  DYVREHKDNI  LYAWEPSFL  NYSVRYRPGL  AYDFLYNYL  TFDYLRSVL  DYLRSVLEDF | A2/A3 supertype  A2  A2/A3 supertype  A2/A26  A2/A26/A3 supertype  A2/A26  A2  A2  A2  A2  A2  A2  A24  A24/A24/A3 supertype  A24  A24  A24  A24  A24  A24  A24  A24  A24 | Jpn J Cancer Res 2001, 92(7): 762-7.  Int J Cancer 2001 94(2): 237-42.  Int J Cancer 2001 94(2): 237-42.  Cancer Res 2001 61(5): 2038-46.  Cancer Res 2001 61(5): 2038-46.  Cancer Res 2001 61(5): 2038-46.  Cancer Res 2001 61(5): 2038-46.  Cancer Res 2001 61(5): 2038-46.  Cancer Res 2001 61(5): 2038-46.  Cancer Res 2001 61(5): 2038-46.  Int J Cancer 2000 88(4): 633-9.  Int J Cancer 2000 88(4): 633-9.  J Immunol 2000 164(5): 2565-74.  Cancer Res 1999 59(16): 4056-63.  Eur J Immunol 2001 31(2): 323-32.  J Urol 2001 166(4): 1508-13.  Prostate 2003 57(2): 152-9.  Eur J Cancer 2004 40(11): 1776-86.  Cancer Res 2001 61(17): 6459-66.  Cancer Res 2001 61(17): 6459-66.  J Immunol 2000 164(5): 2565-74.  Eur J Immunol 2001 31(2): 323-32.  Eur J Immunol 2001 31(2): 323-32. |
| PSMA-624  EZH2-735  PTHrP-102  SART3-511  SART3-734  Lck-90  Lck-449  PAP-248 | PSMA  EZH2  PTHrP  SART3  SART3  p56 ^lck^  p56 ^lck^  PAP | 624-632  735-743  102-111  511-519  734-742  90-99  449-458  248-257 | TYSVSFDSL  KYVGIEREM  RYLTQETNKV  WLEYYNLER  QIRPIFSNR  ILEQSGEWWK  VIQNLERGYR  GIHKQKEKSR | A24  A24  A24  A3 supertype  A3 supertype  A3 supertype  A3 supertype  A3 supertype | Cancer Sci 2003 94(7): 622-7.  Prostate 2004 60(4): 273-81.  Br J Cancer 2004 (91(2): 287-96.  Cancer Immunol Immunother 2007 56(5): 689-98  Cancer Immunol Immunother 2007 56(5): 689-98  Br J Cancer 2007 97(12): 1648-54.  Br J Cancer 2007 97(12): 1648-54.  Clin Cancer Res 2005 11(19 Pt 1): 6933-43. |
